# Comparative safety and efficacy of two bivalent vaccines containing Newcastle disease LaSota and avian influenza H9N2 Sidrap isolate formulated with different oil adjuvants

**DOI:** 10.14202/vetworld.2020.2493-2501

**Published:** 2020-11-24

**Authors:** Jossie Intan Cahyani, Sitarina Widyarini, Michael Haryadi Wibowo

**Affiliations:** 1 Master Program, Faculty of Veterinary Medicine, University of Gadjah Mada, Jl. Fauna No.2, Sleman, Daerah Istimewa Yogyakarta 55281, Indonesia; 2 Pusat Veteriner Farma (Central for Veterinary Biologics), Ministry of Agriculture of the Republic of Indonesia, Jl. Ahmad Yani No.68-70, Ketintang, Gayungan, Surabaya, Jawa Timur 60231, Indonesia; 3 Department of Pathology, Faculty of Veterinary Medicine, University of Gadjah Mada, Jl. Fauna No. 2, Sleman, Daerah Istimewa Yogyakarta 55281, Indonesia; 4 Department of Microbiology, Faculty of Veterinary Medicine, University of Gadjah Mada, Jl. Fauna No. 2, Sleman, Daerah Istimewa Yogyakarta 55281, Indonesia

**Keywords:** avian influenza H9N2 Sidrap isolate, bivalent vaccine Newcastle disease-avian influenza H9N2, Marcol, Montanide ISA70

## Abstract

**Background and Aim:**

Newcastle disease (ND) and avian influenza (AI) are two devastating diseases of poultry, which cause great economic losses to the poultry industry and disrupt food security in our country. The use of ND-AI inactive bivalent vaccine is very effective and economical to prevent and control ND and AI disease. Bivalent ND LaSota-AI H9N2 vaccine is not yet available in Indonesia. The inactivated vaccines used in poultry industry often require oil adjuvant to elicit a sufficient immune response. This study aimed to develop the bivalent inactive vaccines containing ND LaSota and AI H9N2 Sidrap isolate which are local isolates as poultry vaccine candidates, and formulated with two different commercial adjuvants, then compared.

**Materials and Methods:**

Two vaccines bivalent were prepared by emulsifying inactivated Newcastle disease virus (LaSota strain) and AI H9N2 Sidrap isolate viruses with Marcol white mineral oil and Montanide ISA70 adjuvants. Both of bivalent vaccines were tested for safety (physical and histopathological at the injection site) and efficacy in specific-pathogen-free chickens. Parameters used for the evaluation of the efficacy were immunogenicity by hemagglutination inhibition and protection percentage.

**Results:**

Both bivalent vaccines are safe to use. Post-vaccination (PV) immune response was observed using a hemagglutination inhibition test at 2, 3, 4, 5, 6, 7, and 8 weeks of PV. The bivalent vaccine B gives a better immune response to ND at 2, 3, and 4 weeks of PV (p<0.05) compared to the bivalent vaccine A, but in 5, 6, 7, and 8 weeks, the PV does not show differences in the immune response. The immune response to AI H9N2 showed differences at weeks 2 and 3 PV (p<0.05) with the bivalent vaccine B indicated higher immunity. A single immunization with both bivalent vaccines induces 100% protection in chickens that have been vaccinated against the deadly challenge with the virulent ND virus.

**Conclusion:**

Both of bivalent vaccines are safe to use and provide good efficacy against virulent ND viruses, but bivalent vaccine B (with Montanide ISA70 adjuvant) shows better immune response than bivalent vaccine A (Marcol white mineral oil adjuvant).

## Introduction

Newcastle disease (ND) is a highly contagious viral disease, which affects almost all species of domestic and wild birds. ND is a strategic disease in poultry because it can cause losses, including morbidity and mortality up to 100%, decreased meat and egg production, and high eradication costs [1,2]. The disease was first recognized in Indonesia and England in 1926 [3] and ND viruses are now found worldwide. This disease is caused by a virus of genus Avulovirus, subfamily Paramyxovirinae, and family Paramyxoviridae [4]. The ND epidemic in Indonesia first occurred in Java in 1926. The ND case is a serious threat to the poultry industry in Indonesia because this disease is endemic [5,6]. Kencana *et al*. [7] and Wibowo *et al*. [8] stated that ND disease in Indonesia is endemic because there are ND cases throughout the year in various regions that have the potential to cause adverse outbreaks. Until 2019, ND cases were still reported in Sumatra, especially in the provinces of Lampung, South Sumatra, Bengkulu, and Bangka Belitung [9]. Avian influenza (AI) virus infections can cause a variety of symptoms of disease in chickens, from asymptomatic infections to respiratory diseases, accompanied by decreased egg production and symptoms of severe systemic disease with a mortality rate close to 100%. Avian influenza virus (AIV) belongs to the Orthomyxovirus family. Disease severity in poultry is classified as low pathogenic AI (LPAI) and highly pathogenic AI which is determined by the genetic features [10]. AI virus subtype H9N2 is categorized as LPAI virus, but it can cause serious economic losses in poultry industry including reduced egg production and decreased growth rate. The LPAI H9N2 virus was first identified in poultry in the 1960s and spread in Asia in the 1990s [11]. LPAI H9N2 virus infection has been an important risk to the Indonesian poultry industry since the end of 2016. Previously in December 2016, there had been reported cases of disease in laying hens in Sidrap Regency, South Sulawesi, Indonesia. Based on the data of vaccination and clinical symptoms found, such as low percentage of mortality, a very significant decrease of egg quality and production up to 80%, as well as laboratory testing with reverse transcriptase-polymerase chain reaction techniques of HA gene fragment and deoxyribonucleic acid sequencing results, showed a single basic amino acid feature in the cleavage site position. Further phylogenic analysis of HA gene fragment can be concluded that the drop production that occured in poultry layer farms in Sidrap Regency are caused by LPAI virus infection H9N2 subtype [12]. Phylogenic data showed that HA-H9 and NA-N2 were homologous (98%) with Vietnamese H9N2 virus (A/Muscovy duck/Vietnam/LBM719/2014) [13]. Until now, the AI H9N2 virus has spread in many provinces in Indonesia and affected layer, breeder, and broiler chicken farms with the main symptoms of decreased egg production [14]. AIV H9N2 causes weight loss in several broiler farms in Indonesia [13], it has been previously reported that AIV causes clinical disease in broilers so that it can reduce growth rates and adversely affect the feed conversion ratio [15-17].

ND and AI are two devastating diseases of poultry, which cause great economic losses to the poultry industry and disrupt food security. According to the Ministry of Agriculture, Indonesia [18], ND and AI diseases are types of strategic infectious diseases that can threaten and cause enormous economic losses to poultry farms. To resolve this endemic disease, the bivalent inactivated vaccine has been widely used [19]. Bivalent vaccines available in Indonesia are ND-AI bivalent vaccines with H5N1 subtype and AI H5N1-AI H9N2 bivalent vaccine while ND-AI bivalent vaccine with AI H9N2 subtype is not yet available in Indonesia, so it is necessary to develop this bivalent vaccine to prevent ND disease as well as AI H9N2 subtype disease. An effective vaccine needs not only good antigens but also preferable adjuvant to enhance the immunogenicity of antigen. Several studies have showed that inactivated water-in-oil emulsion vaccine is capable of inducing immune response to protect poultry [20-22]. Marcol is one of the mineral oil that is widely used in the manufacture of oil emulsion vaccines [23], but in the manufacture of adjuvant emulsion water in oil (w/o), an emulgator is needed to reduce the interface tension between the oil and water phases and minimize surface energy from droplets that are formed [24]. Montanide ISA70 is a series of Montanide ISA and ready to use adjuvant for water-in-oil (w/o) emulsions that are recommended for use in avian vaccines [25].

Development of bivalent vaccine in this study uses the LaSota ND virus, LaSota can protect against heterologous challenge and it is the most widely used type in the world [26,27] including Indonesia, whereas for AI viruses, it uses the LPAI virus Sidrap isolate which is a local isolate from Indonesia and has never been used for a bivalent vaccine. This study aimed to determine the safety and efficacy of an inactive bivalent ND-AI H9N2 vaccine. Montanide ISA70 adjuvant was used and compared with white mineral oil Marcol adjuvant as an alternative adjuvant. A comparison between two adjuvants mentioned above was evaluated in the difference of safety, immune responses, and protection against virulent ND virus challenge.

## Materials and Methods

### Ethical approval

All animal procedures performed in this study (permit number 0085/EC-FKH/Int./2019) were reviewed, approved, and supervised by the Institutional Animal Care and Use Committee of Veterinary Medical Faculty, Gadjah Mada University.

### Study period and location

The research was conducted from January to May 2020 at Pusat Veteriner Farma (Central for Veterinary Biologics, Ministry of Agriculture, Surabaya, Indonesia) and in the Laboratory of Department Pathology, Faculty of Veterinary Medicine, Gadjah Mada University, Yogyakarta, Indonesia.

### Virus

The ND virus (LaSota strain), AI subtype H9N2 A/chicken/Sidrap/07170094-44O/2017 (certificate of analysis from National Veterinary Drug Assay Laboratory, Ministry of Agriculture, Indonesia), and virulent Newcastle disease virus (NDV) for challenge (10^8^ EID_50_) were obtained from Central for Veterinary Biologics (Pusat Veteriner Farma), Ministry of Agriculture, Indonesia.

### Adjuvants

Montanide ISA70 adjuvant is manufactured by SEPPIC, Paris, France. The Marcol52 white mineral oil adjuvant is the product of ExxonMobil.

### Eggs and chickens

Specific-pathogen-free (SPF) embryonated chicken eggs for virus propagation and inactivation test were used. SPF 3-week-old white Leghorn chickens were used for safety and efficacy test. The eggs for these chickens were certified and purchased from Caprifarmindo Laboratorium Company, Indonesia.

### Preparation of vaccines

#### Vaccine development

Viruses were propagated in the allantois of 10-day-old SPF embryonated chicken eggs for AI H9N2 subtype and ND LaSota. The allantoic fluid was harvested after 72-96 h. The AI-ND virus titers obtained from the propagation was 10^9^ EID_50_ and both viruses are used for antigen production. Harvested material was inactivated by 0.1% formalin. The fluid was blended using magnetic stirrer at 25°C for about 24 h. The absence of inactivated viruses was confirmed by inactivating test in SPF embryonated egg with three passages and the allantoic fluid was tested by a rapid hemagglutination (HA) test [28,29].

#### Antigen emulsification

Two different oil emulsion-inactivated vaccines used in this experiment. The first vaccine or bivalent vaccine A containing the inactivated virus of ND LaSota (hemagglutination/HA activity 2^9^) and AI H9N2 Sidrap isolate (HA activity 2^9^) blended at a ratio 1:1, then mixed with Marcol white mineral oil adjuvant at 30:70 (aqueous phase vol:oil phase vol) and then emulsified using Ultra Turrax. The second vaccine was designated bivalent vaccine B, a homogeny of oil adjuvant of Montanide ISA70 containing the same antigen load with vaccine A and has a ratio 70 oil phase:30 aqueous phase which is used as described by Seppic protocols, respectively. Accelerated physical stability tests were performed by determining the appearance view after storing the emulsions at 37°C for 3 weeks.

### Vaccine evaluation

#### Sterility test

Every virus suspension and an experimental vaccine of the prepared vaccine candidate were tested for sterility and freedom from any fungal or bacterial contaminants by culturing on specific media [28-30].

#### Safety test

Experimental vaccine of the prepared vaccine was tested for its safety by inoculating double dose intramuscular (2×0.5 mL) in ten 3-week-old SPF chickens, and these are observed for 2 weeks for the presence of clinical signs of disease or local lesions [30]. A histopathological examination also performed to see the presence of localized lesions at the injection site[31]. Pectoral muscle tissues (injection site) were fixed with 10% formalin. Thereafter, the samples were processed according to standard procedures employed for hematoxylin-eosin staining [32].

### Efficacy experiment

#### Immunization of animals

Immunization and challenge experiments were performed in accordance with instructions in the OIE and FOHI (Indonesian veterinary pharmacopeia) manual [28-30] to evaluate the effects of different adjuvant. Eighty 3-week-old SPF chickens were randomly divided into two equal groups (bivalent vaccine A with Marcol white mineral oil adjuvant and B with Montanide ISA70 adjuvant) and every group divided again into four groups (A1, A2, A3, and A4; B1, B2, B3, and B4) 10 chickens each. Chickens in Group A1 and A2 were vaccinated intramuscularly with bivalent vaccine A (0.5 mL/chicken) in the breast muscle. The chickens in Groups B1 and B2 were vaccinated with bivalent vaccine B by the same route and dose. Post-vaccination (PV) immune response was monitored using the hemagglutination inhibition (HI) test [28,29] at 2, 3, 4, 5, 6, 7, and 8 weeks PV, serum before vaccination is also collected. Groups A2, A3, B2, and B3 were used for challenge experiment which Groups A3 and B3 were used as controls for the challenge test and were not vaccinated. Chicken group A4 and B4 were control groups that were not vaccinated and did not receive treatment. Blood serum samples for the control group were also collected.

### Challenge of vaccinated chickens with virulent NDV

The challenge experiment was conducted in isolator enclosure at Central for Veterinary Biologics (Pusat Veteriner Farma), Ministry of Agriculture, Indonesia. Two weeks after vaccination, chickens in Groups A2, A3, B2, and B3 were challenged with virulent ND virus 10^8^ EID_50_ through intramuscular. Observations for clinical disease and mortality were carried out for 2 weeks after the challenge.

### Statistical analysis

The comparison of mean serum titers tested by HI was evaluated using analysis of variance and analyzed using software Statistical Package for the Social Sciences version 22 (IBM Corp., NY, USA). Differences were considered significant if p<0.05.

## Results

### Inactivation confirmation of the viruses

To confirm the complete inactivation of the two viruses, formalin-treated viruses were inoculation with three passages in 10-day-old embryonated SPF chicken eggs. All chicken embryos injected with formalin-treated viruses survived for more than 120 h, and no viruses were detected in the rapid HA test.

### Sterility and safety of the prepared vaccines

**Figure-1:**
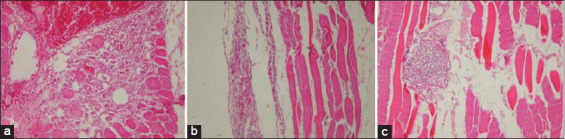
Microscopic muscle tissue in the injection area of bivalent vaccine A (Marcol white mineral oil adjuvant). (a) 3 days post-vaccination (PV), necrosis with lymphocyte and macrophage infiltration; (b) 7 days PV showed lymphocyte; (c) 56 days PV showed lymphocyte follicle focal be encapsulated in vaccination in injection area.

**Figure-2:**
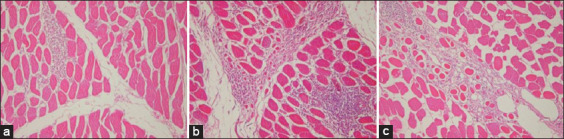
Microscopic examination of the area vaccination injections with bivalent vaccine B (Montanide ISA70 adjuvant); (a) 3 days post-vaccination (PV), mild lymphocyte infiltration is seen; (b) 7 days PV showed lymphocyte infiltration and necrosis; (c) necrosis with lymphocyte infiltration at 56 days after vaccination injection.

All the virus suspension and two vaccine candidates were found to be sterile, where they induced neither any bacterial or fungal growth. Both bivalent vaccines were found to be safe in vaccinated chickens, where no presence of clinical signs of disease or local lesions after double doses inoculation in ten 3-week-old SPF chickens, respectively. Histologically, local lesions were found in injection side tissue samples that were vaccinated by the two bivalent vaccines (Figures-1 and 2). Although histologically there is local inflammation, both bivalent vaccines are still safe to use in chickens because it is a normal response to a vaccine that uses adjuvant.

### Physical stability of the prepared vaccines

An accelerated physical stability test showed that none of both bivalent vaccines tested showed emulsion breakdown after testing.

### Evaluation of immune response by HI test

#### Monitoring of NDV immune response

**Figure-3:**
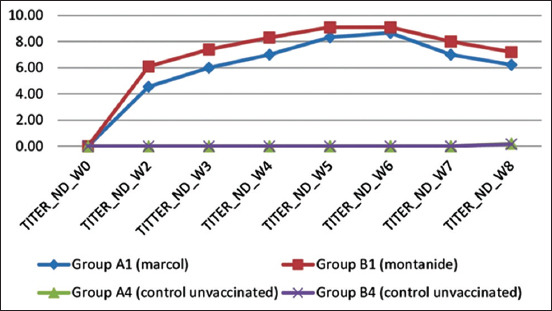
Comparison of hemagglutination inhibition antibody titers against Newcastle disease virus of bivalent vaccine A with Marcol white mineral oil adjuvant and bivalent vaccine B with Montanide ISA70 adjuvant.

Serum sample was collected from vaccinated chickens before vaccination and at 2, 3, 4, 5, 6, 7, and 8 weeks PV and subjected to a HI test. It was noticed that chickens vaccinated with inactive bivalent vaccine A and inactive bivalent vaccine B showed detectable HI antibody titer against ND antigens at 2 weeks PV (≥4 log2) with a mean HI titer 4.5 log2 and 6.1 log2, respectively. As shown in the graph (Figure-3), it is seen that vaccination with a bivalent vaccine B containing Montanide ISA70 adjuvant provides a higher immune response against ND antigens compared to a bivalent vaccine A containing Marcol white mineral oil adjuvant. Data analysis showed that between the bivalent vaccine A and the bivalent vaccine B showed a significant difference (p<0.05) in the HI antibody titer against ND antigens at weeks 2, 3, and 4. However, at weeks 5, 6, 7, and 8, there were no differences in immune response between the two vaccine groups (p>0.05). The highest average HI antibody titer was reached at 6 weeks PV (8.8 log2) for the bivalent vaccine A and 5 weeks PV (9.1 log2) for the bivalent vaccine B.

#### Monitoring of AI H9N2 immune response

**Figure-4:**
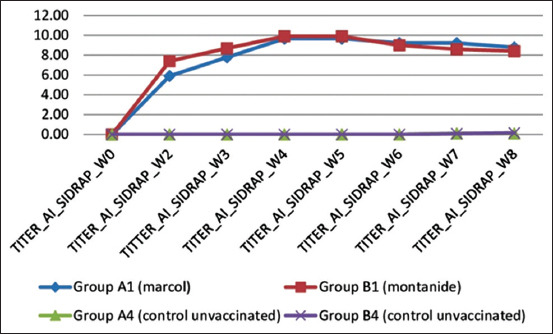
Comparison of hemagglutination inhibition antibody titers against avian influenza virus of bivalent vaccine A with Marcol white mineral oil adjuvant and bivalent vaccine B with Montanide ISA70 adjuvant.

It was noticed that chickens vaccinated with inactivated bivalent vaccine A and inactivated bivalent vaccine B showed increase immune responses against AI H9N2 antigen started at the 2^nd^ week with a mean HI titer 5.6 log2 for vaccine A and 7.4 log2 for vaccine B. The highest titer was observed at 4 weeks PV for both of bivalent vaccine, with a mean HI titer of 9.7 log2 for bivalent vaccine A and 9.9 log2 for bivalent vaccine B. Until the study period ends (8 weeks PV), HI titers were detectable at high level. By comparing the results of adjuvant, on graph (Figure-4), it is noticeable that increase of immune response bivalent vaccine using Montanide ISA70 (bivalent vaccine B) is better than Marcol white mineral oil (bivalent vaccine A) at 2 and 3 weeks PV, data analysis showed significant differences (p<0.05). However, this significant difference disappeared by week 4, 5, 6, 7, and 8 PV.

### Protective effectiveness of prepared vaccines against virulent NDV

**Figure-5:**
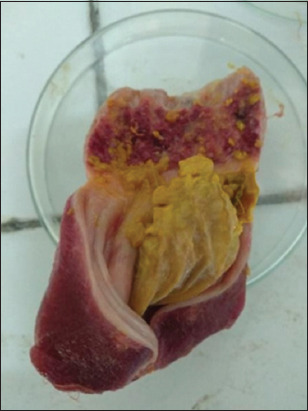
Proventriculus and ventriculus unvaccinated chickens showed ptechiae and hemorrhages.

To evaluate the protective efficiency of both vaccine groups, A2 and B2 vaccinated SPF chicken groups were challenged with virulent NDV at 2 weeks PV. After challenge with virulent NDV, vaccinated chickens with Marcol white mineral oil adjuvant (A2 group) and Montanide ISA70 adjuvant (B2 group) showed no clinical signs of disease and no mortality even after 14 days post-challenge (PC). This test showed that the percent of protection induced by the vaccine candidates that have been prepared is 100% in both bivalent vaccines (Table-1). A rapid increase in the HI titer against NDV after the challenge was observed. The mean HI titer for A2 group was 6 log2 and 9 log2 after the 1^st^ and 2^nd^ weeks PC, while in B2 group, the titers were 7.5 log2 and 9.6 log2, respectively. All unvaccinated control chickens showed typical clinical signs of virulent NDV, and within 4 days PC, 100% mortality rate was observed. Petechiae in proventriculus and ventriculus were observed during postmortem examination which is one of the characteristic lesions of ND infection (Figure-5).

**Table-1:** Immune protection against virulent Newcastle disease virus.

Group	Hemagglutination inhibition antibody titers (log2)			Clinical signs /total	Dead/total	Protection rate* (%)

	Pre-infection	7 days p.c	14 days p.c			
Vaccine A (A2) Marcol	4.6	6	9	0/10	0/10	100
Unvaccinated (A3) control	0^	-	-	10/10	10/10	0
Vaccine B (B2) Montanide ISA70	5.5	7.5	9.6	0/10	0/10	100
Unvaccinated (B3) control	0^	-	-	10/10	10/10	0

Pre-infection=Two weeks PV, p.c=Post-challenge,

* Protection definition: Survival without signs of clinical infection not shedding. ^Unvaccinated control chickens died on day 4^th^ p.c

## Discussion

ND and AI are endemic and very important disease in the world and Indonesia too. Every year, they caused heavy financial loss in the poultry industries [6,8,16]. To prevent these diseases, it is important to produce effective vaccines and ND-AI bivalent vaccine can be used to resolve this problem [19]. ND-AI bivalent vaccine with AI H9N2 subtype is not yet available in Indonesia, so it is necessary to develop the bivalent vaccine to prevent ND disease as well as AI H9N2 subtype disease.

The ND LaSota virus was used because LaSota can protect against heterologous challenge and it is the most widely used strain in the world [26,27] including in Indonesia and AI H9N2 virus that is used for this bivalent vaccine is a Sidrap isolate where this isolate is a local isolate and has never been used as a bivalent vaccine. In developing an inactive bivalent vaccine, adjuvants are needed to improve the immune response. Marcol white mineral oil adjuvant and Montanide ISA70 adjuvant are used in this bivalent vaccine and then compared.

The results of this study indicate that both prepared vaccine candidates were free from foreign contaminants and safe for vaccinating chickens which showed no detectable signs of infection as the recommendation of OIE [28,29]. Histopathological examinations revealed inflammatory lesions that were found in site of vaccination injection All tissue samples were taken on days 3, 7, and 56 PV were vaccinated with vaccine A showed the presence of local inflammatory lesions, as well as the samples vaccinated with vaccine B also showed the same results (Figures-1 and 2). Histological changes at the injection sites of vaccines A and B were also found in previous studies. Long-term observations of the IM or SC DPT vaccine by Verdier *et al*. [33] report that adjuvant is maintained for 9-12 months at the vaccination site. As is well known, this can induce inflammatory nodules that persist for several months without any pathological signs at the injection site. In the Cervarix vaccine (HPV vaccine), inflammatory nodules are also present at the site of vaccination after 1 month of vaccination which is identified as macrophage nodules [34]. Histopathological observations of the bivalent A vaccine and the bivalent B vaccine on 3, 7, and 56 days after vaccination still show an inflammatory reaction up to 56 days of PV (the end of this study), in line with what was reported by Kashiwagi *et al*. [35]. The use of adjuvants causes an inflammatory response but it is limited to where it is injected, is not systemic and the nodules persist for several months [35].

Congenital immunity consists of two patterns, namely, pathogen-associated molecular patterns (PAMPs) and damage-associated molecular patterns (DAMPs). PAMPs recognize microbial components (bacterial and viral products) and other endogenous products released from damaged cells (damage signals) and then these signals stimulate DAMPs that activate inflammation [36]. According to Oreskovic *et al*. [37], adjuvants can also affect the balance between the production of specific antibodies with antigens and cellular immune responses. However, the use of different adjuvants can cause undesirable local reactions. This can be time-dependent, as demonstrated by the opposite dendritic (DC) cell activation between oil-based adjuvants and Al(OH)3, and also by chemokines and cytokine expression during 4 and 24 h of PV [38]. Whereas oil emulsion and Montanide ISA provide a brief local reaction but still provide an adequate immune response with activation of DC in the skin, this leads to Th1 and Th2 responses [37]. According to both macroscopic and microscopic data obtained from our study it can be concluded that our bivalent vaccine A with adjuvant Marcol white mineral oil and bivalent vaccine B with adjuvant Montanide ISA70 based on oil emulsion can be used as a safe vaccine.

A perfect oil adjuvant should enhance the immune response and reduce the number of immunization required. The efficacy test was also carried out in this study to determine the effectiveness of the two bivalent vaccines using Marcol white mineral oil adjuvant and Montanide ISA70 adjuvant, respectively. Serum samples were taken before vaccination and 2, 3, 4, 5, 6, 7, and 8 weeks after vaccination to measure HI antibody titers against ND and AI H9N2. The two formulated bivalent vaccines using Marcol white mineral oil adjuvant (bivalent vaccine A or A1 group) and Montanide ISA70 (bivalent B vaccine or B1 group) developed a protective immune response against ND and AI H9N2 in vaccinated SPF chickens by weeks 2 PV using a single dose, as in research conducted by Liu *et al*. [25] and Zhao *et al*. [22] which state that the NDV and AIV H9N2 vaccines that use both oil adjuvants are able to increase antibody titers.

In this study, although both vaccines were equally capable of inducing an immune response, the HI antibody titer against ND in vaccines using Montanide ISA70 at weeks 2, 3, and 4 PV showed a significantly higher antibody titer (p<0.05) than with a vaccine that uses Marcol white mineral oil. A significant increase in antibody titer against ND in vaccines using adjuvant Montanide ISA70 at weeks 2, 3, and 4 was also developed by Jafari *et al*. [39]. Peak levels of HI antibodies were detected at 6 weeks PV for vaccine using Marcol white mineral oil with a mean HI titer of 8.8 log2 and at 5 weeks PV for vaccine using Montanide ISA70 with a mean HI titer 9.1 log2 against NDV, as shown in Figure-3.

For HI antibody titers against AIV H9N2, statistically, the results showed that vaccine with Montanide ISA70 had significantly higher antibody titers at weeks 2 and 3 PV when compared with vaccine using Marcol white mineral oil (p<0.5). Vaccine A which containing Marcol adjuvant showed an increase in the immune response starting at week 2 (6 log2) and reached a peak at week 4 (9.7 log2), and keep the high titer up to 5 weeks. Starting by the 6 week, the titer gradually decreased to the end of the study at week 8 PV, but the titer still showed high antibody titers of 8.8 log2 (Figure-4). The previous studies have shown that vaccine emulsified with Marcol adjuvant showed an increase in the immune response to AI H9N2 at week 2 with peak at week 4 (9.5 log2), which then declined gradually starting at week 5 [22]. Vaccine B which using Montanide ISA70 adjuvant showed peak levels of HI antibodies at 4 weeks PV with a mean HI titer of 9.9 log2, this number persisted until the following week and began to decline gradually at week 6 PV.

Choi *et al*. [40] and James *et al*. [41] stated that vaccine emulsions prepared with adjuvant Montanide ISA70 provide good protection not only inducing a humoral immune response but also a strong cellular immune response. Liu *et al*. [25] stated that ISA70 Montanide adjuvant could be the best adjuvant as the best adjuvant in animal vaccine production, which in his research showed that Montanide ISA70 provides higher HI antibody titer compared to mineral oil adjuvant during research with highest antibody titer 10.7 log2 for Montanide ISA70 and 8.9 log2 for mineral oil at the 4^th^ week after vaccination. Montanide ISA70 adjuvant provides better immune response results compared to Marcol white mineral oil adjuvant because Montanide ISA70 has better viscosity than white mineral oil Marcol, therefore, vaccine is not easily diffuse. Montanide ISA70 does not need an emulgator because of its better viscosity so that it can induce the immune response continuously, while white mineral oil requires an emulgator to reduce the interface tension between the oil and water phases so it is not diffuse easily [24]. The vaccine produced with Montanide ISA has good viscosity so it has several advantages such as being able to increase the number of T lymphocytes, macrophages and has a good exposure effect between antigens and cells or certain proteins in the immune system [42].

The pathogenesis of NDV and AIV H9N2 can be aggravated due to the presence of secondary agents such as environmental factors and other pathogenic coinfections [43,44]. For proper NDV and AIV control in endemic countries, not only effective mass vaccination strategies are needed but also a focus on using potent vaccines that are capable of inducing an early PV immune response.

To evaluate the protective efficacy of both vaccine groups, SPF chickens that have been vaccinated with bivalent vaccine A (A2 group) and bivalent vaccine B (B2 group) and SPF chickens that not vaccinated (A3 and B3 group) are challenged with virulent ND virus in the 2^nd^ week PV. Observations were made in the Central for Veterinary Biologics (Pusat Veteriner Farma) isolator enclosure for 14 days. Challenge results showed severe clinical signs with 100% mortality in the control unvaccinated group PC. All non-vaccinated chickens died after 4 days PC. Control chickens that died were performed necropsy on the proventriculus and ventriculus to see postmortem changes due to virulent NDV (Figure-5). Postmortem examination of the dead birds revealed characteristic lesions of ND such as petechiae in the proventriculus, white punctate necrosis in the spleen, and hemorrhages in the intestine and cecal tonsil [22]. Chicken groups that were vaccinated and then challenged with virulent NDV showed 100% protection, without any mortality and clinical signs of NDV infection (Table-1). This fulfills the OIE and FOHI [28,30] requirement concerning the evaluation of vaccines by challenge test as any vaccine candidate must show percentage protection value at least 90% during 14 days challenge period.

Allan *et al*. [45] reported 100% of deaths due to challenges with virulent ND viruses when HI titer was 2 log2 or less. Otherwise there were no deaths when HI antibody titers of birds were between 4 log2 and 6 log2 with an average of 5.2 log2 HI antibody titer. The Results of the challenge test of this study are in line with the previous reports. HI titers against ND virus before being infected with virulent ND virus were 4.4 log 2 for A vaccine and 5.5 log 2 for B vaccine. In this study, HI NDV titer of 4 log2 was considered protective. Our result supported by the previous study that reported chickens with HI 4 log2 titers protected from challenges with a virulent ND virus [46].

## Conclusion

Both of prepared bivalent vaccine candidates are safe for vaccination in chickens and are both able to induce an immune response against NDV and AIV H9N2 but the bivalent vaccine B containing Montanide ISA70 adjuvant provides a better and higher immune response than the bivalent vaccine A containing Marcol white mineral oil adjuvant. Bivalent vaccine A and bivalent vaccine B are both able to provide good protection against ND virus challenge with 100% protection in SPF chickens. Both of vaccines can be used in the poultry industry to prevent and control ND and AI H9N2 diseases in chickens in Indonesia.

## Authors’ Contributions

JIC conceptualized, designed, and planned the aim of the study. MHW and SW supervised the experiments and corrected the manuscript. All authors read and approved the final manuscript.
